# Targeting BCL6 and STAT3 in triple negative breast cancer: the one-two punch?

**DOI:** 10.18632/oncoscience.270

**Published:** 2015-11-21

**Authors:** Sarah R. Walker, David A. Frank

**Affiliations:** Department of Medical Oncology, Dana-Farber Cancer Institute and Departments of Medicine, Brigham and Women's Hospital and Harvard Medical School, Boston, MA; Department of Medical Oncology, Dana-Farber Cancer Institute and Departments of Medicine, Brigham and Women's Hospital and Harvard Medical School, Boston, MA

**Keywords:** STAT3, BCL6, triple negative breast cancer

Breast cancer remains the second leading cause of cancer deaths in women in the United States. Triple negative breast cancer, tumors lacking estrogen receptor, progesterone receptor, and Her2, only comprise about 20–30% of breast tumors diagnosed. However, due to the lack of specific targeted therapy as exists for ER+ or Her2+ tumors, they account for many of the breast cancer deaths. To develop targeted therapy for triple negative breast cancer, inhibiting two targets may be necessary.

One new target of interest is the transcriptional modulator BCL6 which has been recently identified as playing an important role in breast cancer [1–3]. BCL6 has been characterized as a transcriptional repressor that recruits various corepressor complexes to repress its target genes; however, genes have also been identified that are upregulated by BCL6 [1, 2], including Zeb1 which is involved in promoting EMT [3]. While the roles of BCL6 in preventing terminal differentiation of B cells in the germinal center and promoting diffuse large B cell lymphoma are well known, little is currently known about the roles of BCL6 in solid tissue. BCL6 is amplified in ∼50% of breast tumors and is expressed in most breast cancer cell lines, including triple negative breast cancer cell lines [1]. Furthermore, BCL6 expression is correlated with disease progression and poor overall survival [2], and targeting BCL6 results in reduced growth and loss of breast cancer cell viability [1]. Importantly, triple negative breast cancer cell lines were among the most sensitive to BCL6 inhibition. In the normal mammary gland, BCL6 has been shown to prevent terminal differentiation and milk production, in part due to competition with signal transducer and activator of transcription 5 (STAT5) for regulation of target genes [4].

STATs are latent transcription factors that remain in the cytoplasm until activated by tyrosine phosphorylation often via Jak kinases. They then translocate to the nucleus as an active dimer and modulate transcription of target genes. There are seven members of the STAT family and four members, STAT1, STAT3, STAT5a, and STAT5b regulate the expression of BCL6. While STAT1 and STAT3 upregulate BCL6 expression, STAT5 (5a and 5b) downregulates BCL6 expression [5]. All four STATs have been shown to play roles in breast cancer; however, STAT3 activation has been linked to more aggressive types. While STAT3 activation can occur in any subtype of breast cancer [5], STAT3 activation is restricted largely to triple negative breast cancer cell lines, and STAT3 signaling has been shown to be important for the survival of triple negative breast tumors [6].

Both BCL6 and STAT3 play critical roles in triple negative breast cancer, including promoting survival and EMT, through modulating largely distinct target genes. Thus, could targeting these two factors together be a useful strategy for specifically treating triple negative breast cancer? Inhibition of BCL6 by siRNA or the peptidomimetic RI-BPI [7] in conjunction with inhibition of STAT3 with the Jak kinase inhibitors TG101348 or nifuroxazide led to enhanced killing of triple negative breast cancer cell lines [1]. These findings suggest that targeted therapy directed simultaneously towards two oncogenic transcriptional modulators may be a new avenue for the treatment of triple negative breast cancer.
